# Study protocol for a randomized controlled trial comparing mindfulness-based cognitive therapy with maintenance anti-depressant treatment in the prevention of depressive relapse/recurrence: the PREVENT trial

**DOI:** 10.1186/1745-6215-11-99

**Published:** 2010-10-20

**Authors:** Willem Kuyken, Sarah Byford, Richard Byng, Tim Dalgleish, Glyn Lewis, Rod Taylor, Edward R Watkins, Rachel Hayes, Paul Lanham, David Kessler, Nicola Morant, Alison Evans

**Affiliations:** 1 Mood Disorders Centre, School of Psychology, University of Exeter, EX4 4QG, UK; 2 King's College London, Centre for the Economics of Mental Health, Box PO24, Institute of Psychiatry, De Crespigny Park, London, UK; 3 Peninsula College of Medicine and Dentistry, University of Plymouth, Plymouth, UK; 4 Medical Research Council Cognition and Brain Sciences Unit, Chaucer Road, Cambridge, UK; 5 Academic Unit of Psychiatry, University of Bristol, Cotham Hill, UK; 6 c/o Depression Alliance, 20 Great Dover Street, London, UK; 7 Dept. of Social and Developmental Psychology, University of Cambridge, Cambridge, UK

## Abstract

**Background:**

Depression is a common and distressing mental health problem that is responsible for significant individual disability and cost to society. Medication and psychological therapies are effective for treating depression and maintenance anti-depressants (m-ADM) can prevent relapse. However, individuals with depression often express a wish for psychological help that can help them recover from depression in the long-term. We need to develop psychological therapies that prevent depressive relapse/recurrence. A recently developed treatment, Mindfulness-based Cognitive Therapy (MBCT, see http://www.mbct.co.uk) shows potential as a brief group programme for people with recurring depression. In two studies it has been shown to halve the rates of depression recurring compared to usual care.

This trial asks the policy research question, is MBCT superior to m-ADM in terms of: a primary outcome of preventing depressive relapse/recurrence over 24 months; and, secondary outcomes of (a) depression free days, (b) residual depressive symptoms, (c) antidepressant (ADM) usage, (d) psychiatric and medical co-morbidity, (e) quality of life, and (f) cost effectiveness? An explanatory research question asks is an increase in mindfulness skills the key mechanism of change?

**Methods/Design:**

The design is a single blind, parallel RCT examining MBCT vs. m-ADM with an embedded process study. To answer the main policy research question the proposed trial compares MBCT plus ADM-tapering with m-ADM for patients with recurrent depression. Four hundred and twenty patients with recurrent major depressive disorder in full or partial remission will be recruited through primary care. Depressive relapse/recurrence over two years is the primary outcome variable. The explanatory question will be addressed in two mutually informative ways: quantitative measurement of potential mediating variables pre/post-treatment and a qualitative study of service users' views and experiences.

**Discussion:**

If the results of our exploratory trial are extended to this definitive trial, MBCT will be established as an alternative approach to maintenance anti-depressants for people with a history of recurrent depression. The process studies will provide evidence about the effective components which can be used to improve MBCT and inform theory as well as other therapeutic approaches.

**Trial registration number:**

ISRCTN26666654

## Background

Depression is a major public health problem that, like other chronic conditions, tends to run a relapsing and recurrent course [1], producing substantial decrements in health and well-being [2]. WHO predicts that by 2020 depression will be the second leading cause of disability in the world [3]. The current cost of mood disorders in the UK has been calculated at 1.5% of GDP [4] and a recent King's Fund report projects that the cost will be £12.15 billion by 2026 [5]. More than 50% of patients experience at least 2 episodes of depression. Moreover, without ongoing treatment people suffering recurrent depression suffer relapse/recurrence at rates as high as 80%, even after successful acute treatment [6,7]. Thus, most of the prevalence, burden and cost of depression is a consequence of relapse/recurrence and the majority of the burden attributable to depression could be offset through interventions aimed at the prevention of depressive relapse/recurrence [8]. Currently the majority of depression is treated in primary care, and m-ADM is the mainstay approach to preventing relapse/recurrence [9-11]. To stay well NICE recommends that people with a history of recurrent depression continue m-ADM for at least two years [11]. However, many patients experience unpleasant side-effects, rates of ADM adherence tend to be low, and many patients express a preference for psychosocial interventions [12-14]. Service user organisations therefore advocate greater availability of psychosocial therapies. Similarly, the King's Fund recommends: "expansion of evidence-based interventions in primary care" and "more research into the cost-effectiveness of a range of interventions, including mental health promotion and prevention initiatives" [[5]; p. xxi]. In line with this, significant government initiatives such as the Improving Access to Psychological Therapies programme are beginning to explore accessible, acceptable and cost-effective psychosocial models of care and envisage up to 250 centres offering psychosocial treatments within a decade [4].

### Psychosocial approaches to prevent depressive relapse/recurrence

While there is a strong evidence base for psychosocial treatment of current depression, only more recently has attention turned to preventing depressive relapse/recurrence. Policy initiatives, user group and professional consensus recommend as priorities for future research the development of psychosocial interventions to prevent depressive relapse/recurrence and the use of non-traditional delivery systems, such as group interventions, to maximise accessibility and cost-effectiveness [15,16]. MBCT is a psychosocial group-based relapse prevention programme. It was developed from translational research into mechanisms of depressive relapse/recurrence [17]. There is much clinical enthusiasm for MBCT, as evidenced by high rates of patient engagement and the recent establishment of MBCT therapist training programmes in the UK at the Universities of Bangor, Exeter and Oxford. In summary, MBCT shows the potential to contribute significantly to reducing the prevalence of depression in UK primary care settings.

### Secondary research findings

Narrative reviews of the broad class of mindfulness-based approaches suggest that they produce substantial improvements on measures of depressive symptoms compared with control groups: Cohen's d (or standardised mean difference) = .86, SD = .3 [18]; Cohen's d = .54, 95% CI .39-.68 [19]. Two uncontrolled trials of patients with a history of recurrent depression and high levels of residual symptoms demonstrate improvements in depressive symptoms, with many people in remission at follow up [20,21]. The two most significant well controlled MBCT randomised controlled trials to date found that MBCT plus usual care halved rates of relapse compared to usual care alone, over sixty weeks of follow-up [22,23]. However, both trials excluded people receiving the current treatment of choice (m-ADM), did not compare MBCT with another active treatment, were not based in real world healthcare settings, had relatively short follow-ups, and did not speak to MBCT's mechanism of change. A recent systematic review of the few existing MBCT controlled trials for depression (including these 2 randomised controlled trials) suggests a significant additive effect of MBCT over usual care for patients with recurrent depression, but only for patients who have experienced three or more previous episodes [24]. However, the authors of this review found no trials comparing MBCT with an active treatment, and suggest this as the next step. Moreover, they found no evidence of the "specific effectiveness of MBCT, despite this being the logical progression from the current research." That is to say, no trials speak to whether MBCT works through its specific hypothesised mechanisms and/or through non-specific cognitive-behavioural, psycho-education and group/therapist support components. They suggest "the need for randomised controlled trials to compare MBCT with other non-pharmacological approaches" and that include tests of the specific and non-specific mechanisms of change [24]. A key issue that the current literature leaves unresolved is whether the robustly demonstrated relapse prevention effects of MBCT are due to the hypothesized mechanism of change, the cultivation of mindfulness skills.

### Our exploratory trial

In our exploratory trial 123 patients with recurrent depression on m-ADM were randomised to either continued m-ADM or MBCT plus m-ADM tapering/discontinuation [25]. The findings suggest that MBCT may not only provide an alternative to m-ADM (relapses at 15 months: MBCT 47% *vs*. m-ADM 60%), but that in an adequately powered definitive phase III trial it may produce superior outcomes. The study suggested that MBCT was also superior to m-ADM in terms of improved quality of life, reduced residual depressive symptoms, and reduced psychiatric co-morbidity. Finally, a secondary qualitative study suggested several putative mechanisms of action [26].

### Why is this trial needed now?

First, we have sufficient evidence from existing trials and our exploratory trial to progress to the next stage of the treatment development process: a definitive randomised controlled trial. That is to say, there is preliminary evidence suggesting that MBCT has potential to significantly reduce the prevalence of depression and to do so cost-effectively. Second, in the UK the vast majority of depression presents in primary care, yet the studies reviewed above do not speak to the generalisability of MBCT to real world primary care settings. Third, the randomised controlled trials to date [22,23] were conducted by the group that developed MBCT and an independent replication is needed. Fourth, depression relapse prevention trials are needed that use a more sophisticated and patient-centered approach to outcome assessment that extends beyond 1-year follow-up and assesses patient-centred secondary outcomes [27]. Fifth, none of the research to date speaks to whether MBCT is efficacious through its hypothesised mechanism of action. Such mechanisms research informs theory and treatment and may produce a simpler and more cost-effective approach to relapse prevention. Finally, the ISRCTN Register (June, 2008) records no comparable recent or ongoing trials in the UK. No current trials of recurrent depression speak to mechanisms of change.

## Methods/Design

### Design

The design is a single blind, parallel RCT examining MBCT vs. m-ADM with an embedded process study. To answer the main policy research question: "Is MBCT superior to maintenance antidepressant medication (m-ADM) in preventing depression over 24 months?" the proposed trial compares MBCT plus ADM-tapering with m-ADM for patients with recurrent depression. Depressive relapse/recurrence over two years is the primary outcome variable.

The trial will answer the explanatory question "Is an increase in mindfulness skills the key mechanism of change?" in two mutually informative ways: quantitative measurement of potential mediating variables pre/post-treatment [28] and an embedded qualitative study to elicit service users' experiences of treatment.

### Setting, participants, recruitment and randomization

Four hundred and twenty patients will be recruited through primary care and treated in accessible primary care or community settings. Inclusion and exclusion criteria were refined through the exploratory trial to maximise real world applicability to the population of people in primary care with recurrent depression who are treated with ADM and who are interested in considering a psychological approach to relapse/recurrence prevention. Inclusion criteria are: a diagnosis of recurrent major depressive disorder in full or partial remission according to the DSM-IV, with 3 or more previous major depressive episodes; aged 18 or older; and on a therapeutic dose of ADM in line with the British National Formulary (BNF) and NICE guidance [11]. Currently MBCT is indicated only for more recurrent depression (3 or more episodes) based on the initial trial and its procedural replication [24]. To meet inclusion criteria the participant must have experienced three previous episodes where depression is the primary disorder and not secondary to substance abuse or bereavement. Exclusion criteria are: patients who are currently depressed, co-morbid diagnoses of current substance abuse (patients with previous substance abuse are eligible for inclusion as long as they are in sustained full remission); organic brain damage; current/past psychosis, including bipolar disorder; persistent antisocial behaviour; persistent self-injury requiring clinical management/therapy; and formal concurrent psychotherapy. We include older adults with depression on the basis of the high prevalence of depression in older adults and a promising pilot study of MBCT with this group [29].

The exploratory trial developed a recruitment methodology that proved acceptable and effective [30]. In each of the four localities (Bristol, Exeter, Plymouth/South Devon and North Devon) and five compact 'time slices' that coincide with MBCT group times, research staff will recruit participants meeting inclusion and exclusion criteria, attempting to ensure that baselines assessments occur as closely as possible, normally within one month, to the start of the next MBCT group.

***Randomisation ***will use permuted block randomisation using computer generated quasi-random numbers. Randomisation will be stratified according to recruitment locality (4 sites, enabling a steady flow to treatment groups) and participants' symptomatic status at intake assessment (asymptomatic *vs*. partially symptomatic), using the GRID-Hamilton Rating Scale for Depression GRID-HAMD [31] cut-off of less than eight being asymptomatic and greater than or equal to 8 being partially symptomatic.

**Figure 1:**
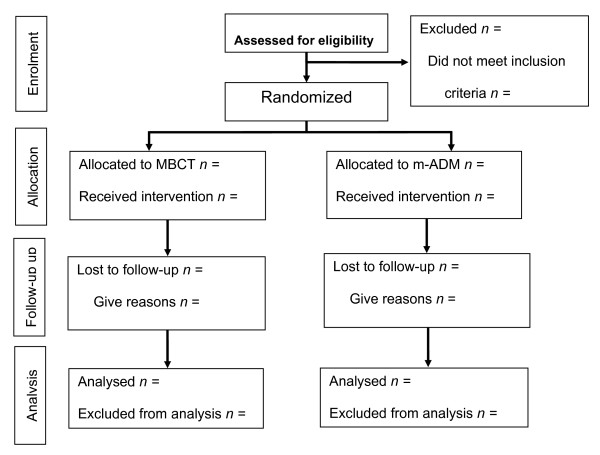
**Consort diagram**.

Figure 1 summarises the trial Consort diagram.

### Interventions

#### Maintenance antidepressants (m-ADM)

As part of the inclusion criteria, patients eligible to take part must be receiving treatment with antidepressant medication in line with the BNF and 2009 NICE guidelines. The m-ADM relapse/recurrence prevention intervention will be an extension of the ADM treatment that is an inclusion criterion for the study. In the UK almost all ADM prescriptions are issued by GPs [32]. Therefore GPs will be responsible for patients' ADM treatment in line with NICE guidelines and contemporary best practice [11]. NICE recommends that patients be maintained on ADM for 2 years after achieving remission, and then should only be tapered in accordance with the NICE guidance for recurrent depression. The guideline recommends that factors such as age, co-morbidity and other risk factors should inform the use of longer-term m-ADM. In the exploratory trial, at study entry the mean time on m-ADM was 340 days, with a significant distribution of patients who had been taking m-ADM for many years. The NICE guidance also speaks to monitoring, ADM type, dose, switching and augmentation.

Medication adherence will be monitored through patients' self-report using the Adult Service Use Schedule (AD-SUS) [33] and the Morisky Medication Adherence Instrument [34] and through manual checks of the GP practice databases at 12 and 24 month follow-ups. We will encourage patients to adhere to medication for the full length of the trial by writing to both the patient and their GP explaining that they have been randomised to maintenance antidepressants and asking them to continue to take a therapeutic level of antidepressants for the 2-year duration of the trial.

***Mindfulness-based Cognitive Therapy (MBCT, see ***http://www.mbct.co.uk**) **is an 8-week, group based programme (12-15 patients per group), designed to teach skills that prevent depressive relapse/recurrence. It is a fully manualised psychosocial intervention with the treatment rationale for each session outlined in full [17]. It is derived both from mindfulness-based stress reduction, a programme with demonstrated efficacy in ameliorating distress in people suffering chronic disease [35], and from cognitive-behavioural therapy for acute depression [36], a programme with demonstrated efficacy in preventing depressive relapse/recurrence [37]. MBCT is based on theoretical and empirical work showing that depressive relapse is associated with the reinstatement of automatic modes of thinking, feeling and behaving that are counter-productive in contributing to and maintaining depressive relapse and recurrence (e.g., self-critical thinking and behavioural avoidance) [38]. Participants learn to recognize these "automatic pilot" modes and to deal with them in more functional ways by employing complementary cognitive-behavioural and mindfulness techniques. The cognitive-behavioural component involves responding to negative thinking and behavioural activation. In the latter stages of the course patients develop an "action plan" that sets out strategies for responding when they become aware of early warning signs of relapse/recurrence. The mindfulness component involves extensive rehearsal of mindfulness skills (e.g., meditation practice) designed to improve patients' attentional control, ability to decentre from negative thinking, and emotion regulation. Session content includes psycho-education, teaching/discussion of key cognitive-behavioural skills, guided mindfulness practices, review of weekly homework (40 minutes of mindfulness practice per day and generalisation of cognitive-behavioural skills). MBCT is an accessible and acceptable treatment as evidenced by low attrition in trials and experience in a range of clinical settings.

The MBCT therapists will be mental health professionals with extensive training in MBCT. All therapists will be judged as ready to instruct MBCT classes by an MBCT expert external to the trial prior to starting the trial groups. During the trial, therapists will receive weekly supervision from an experienced MBCT therapist with the requisite skills to supervise MBCT.

In our exploratory trial we developed a model for offering MBCT in primary care settings to groups of up to 15 patients, with built in checks on accessibility/acceptability, therapist adherence, and competence. Eighty-five per cent of people randomised to MBCT participated in ≥4 sessions and mean ratings of MBCT acceptability were high. Levels of MBCT instructor competence and adherence in the exploratory trial were judged to be at or above acceptable levels by an MBCT therapist independent of the trial [25]. An MBCT therapist independent of the trial will provide checks on adherence/competence based on video-taped MBCT sessions. Adherence will be assessed using the Mindfulness-based Cognitive Therapy Adherence Scale [MBCT-AS; [39]]. The MBCT-AS scale is a 17-item measure of observable therapist behaviours (rated 0, *no evidence*; 1, *slight evidence*; 2 *definite evidence*; range of scores 0-34).

##### ADM tapering in the MBCT arm: Rationale and protocol

The rationale for this trial is the development of a psychosocial intervention for the prevention of depressive relapse/recurrence that provides an *alternative *to the current treatment of choice, m-ADM. Consequently, once patients in the MBCT arm of the trial have learned skills for preventing relapse/recurrence, they will be invited to taper ADM with GP support. A protocol for ADM tapering was developed in the exploratory trial in accordance with NICE guidance regarding information provided to the patient, time course, monitoring of discontinuation/withdrawal symptoms, and consideration of the drugs half-life. In the exploratory trial over the follow-up period (*circa *450 days), the mean number of days on ADM was significantly different across the two groups: m-ADM, M = 411.4 > MBCT, M = 266.46; t = 5.40, *p *< 0.0001. Six months after the end of MBCT 75% of the patients in the MBCT arm of the trial had successfully discontinued their medication. These data testify to the relative success of the tapering/discontinuation regime established in the exploratory trial. However, they also highlight the pragmatic reality that some GPs and/or patients will choose not to taper and we have therefore introduced ADM use as a secondary outcome and the analysis plan will examine this variable in the primary and secondary analyses. We will encourage patients to discontinue their medication within 6 months of the end of their MBCT group.

The MBCT manual has been adapted to include more work on developing a relapse signature and response plan that explicitly includes discontinuation/resumption of ADM. If patients in the MBCT arm experience a significant deterioration following tapering, they will be encouraged to use the skills they learned in MBCT.

### Sample size

Different relapse prevention interventions with different populations produce different absolute rates of depressive relapse/recurrence. Therefore, we have based our sample size on estimated hazard ratios for MBCT *vs*. m-ADM rather than estimated absolute relapse/recurrence rates. We canvassed service users and clinicians who concurred that a relative reduction in relapse/recurrence of 10% would be clinically important. ***(i) The policy question: ***The systematic review of MBCT *vs*. usual care for patients with recurrent depression reports hazard ratios of .47 and .28 for the key trials [24]. We applied several conservative assumptions (first row of Table 1). First, even though the exploratory trial data suggest that the hazard ratio was improving in favour of MBCT as the length of follow-up increased [25], we assumed the hazard ratio of .63 at 15 months to power the proposed trial at 24-months follow up. Second even though attrition from MBCT trials to date is consistently <15%, we have assumed an attrition rate of 20% over the 24-months of follow-up. Finally, we assumed there may be a small clustering effect (ICC = 0.01). The resultant sample size calculation assumptions and output are shown in Table 1 based on 80% power and significance set at .05. This leads to a total sample size of 420 across the two groups.

**Table 1:** Sample size calculation

Author	Comparison	Mean hazard ratio	ICC	Design factor**	Attrition rate	**Sample size per group**^**a**^
Conservative scenario	MBCT *vs*. m-ADM	0.63	.01	1.11	20%	210

Kuyken et al., 2008 [25]	MBCT *vs*. m-ADM	0.63	-0.02	1.0	7%^a^	160

Ma & Teasdale, 2004 [22]	MBCT *vs*. usual care	0.28	-0.008	1.0	3%^b^	14^c^

Teasdale et al., 2000 [23]	MBCT *vs*. usual care	0.47	-0.04	1.0	5%^b^	41^c^

Sample size necessary for 90% power at 5% significance at 24 months;

^a ^at 15 months; ^b ^at 60 weeks, ^c^assuming exponential survival function.

Note group size of 12

***For the secondary outcomes***, meta-analyses of generic mindfulness approaches suggest medium effect sizes in changes in depressive symptoms [19,40] and our exploratory trial suggests medium effect sizes for the secondary outcomes of residual depressive symptoms, psychiatric co-morbidity and quality of life [25]. The sample size estimate for our policy question will enable us to detect a medium between-groups effect size (standardised mean difference or Cohen's d = 0.40) on the main secondary outcomes.

### Proposed outcome measures

The primary outcome in depression relapse prevention studies is sustained remission (i.e., freedom from relapse/recurrence). In addition, patients, health care professionals and NHS policy makers value other outcomes including quality of life, depression free days and co-morbidities. There was preliminary evidence from our exploratory trial that MBCT may prove to be superior to m-ADM on these primary and secondary outcomes.

#### Primary outcome

The occurrence of any depressive relapse/recurrence, and time from randomisation to relapse/recurrence, will be assessed using the Longitudinal Interval Follow-up Evaluation, a form of the Structured Clinical Interview for DSM-IV (SCID) [41,42] designed for longitudinal studies of depression. A patient will be judged to have had a relapse/recurrence if s/he was diagnosed as having a major depressive episode (a score of 5 for 2 consecutive weeks) at any time during the 24-month follow-up period.

#### Secondary outcomes

A unique aspect of our trial is the inclusion of a range of secondary outcome measures including those highly valued by patients themselves. Residual depressive symptoms will be assessed with the observer-rated interviewer administered 17-item HRSD [43] and a well established self-report measure, the [Beck Depression Inventory, 2nd edition; [44]]. Psychiatric co-morbidity will be assessed with the full SCID, and medical co-morbidities with a screening questionnaire. Depression free days will be calculated from the SCID. Quality of life will be assessed with the EQ-5D health-related quality of life measure [45,46].

#### Process data

Our explanatory question asks "is an increase in mindfulness skills the key mechanism of change of MBCT?" We address this via embedded quantitative and qualitative process-outcome studies across the trial arms. Quantitative measures will be administered at baseline and again one month after the end of the MBCT (or the equivalent time point in the m-ADM arm) so that change scores can be used to predict treatment outcome at the different follow ups using mediational analyses [28]. Critically, this design ensures that changes in putative mediators temporally precede the primary outcome (relapse/recurrence), a prerequisite in inferring mediation, and allows baseline-to-post-treatment change in symptoms to be statistically controlled. The same measures will be administered at study end to assess stability of change. We will also use experience sampling methods on sub-samples at study end to test emerging models of emotion regulation post-MBCT. All these assessments will comprise validated questionnaire and cognitive-experimental measures of the variables that we hypothesise mediate MBCT's effects.

In addition, qualitative data will be collected via semi-structured interviews and written responses to access patients' own accounts of the mechanisms and impacts of treatment. At the end of treatment (or the equivalent time point in the m-ADM arm), all participants will write short accounts of their experiences of and perceived impacts of treatment in response to open-ended questions. We will collect similar data from all participants at the end of the trial in order to gain a broad understanding of the impacts of both MBCT and ADM tapering and m-ADM over the follow-up period. Additionally at trial end, semi-structured interviews designed to obtain a more in-depth understand of the ongoing mechanisms and impact of treatment over the follow-up period will be conducted with sub-samples. Interviews will focus on the patients' views of the role of mindfulness, strategies to prevent or cope with relapses, and broader impacts of treatment in patients' lives, and will be informed by previous work on experiences of MBCT [26]. Integration with the quantitative process data will enhance understanding of change mechanisms that can improve MBCT's potential efficacy.

### Economic evaluation

Economic evaluation will be carried out by the trial health economist (SB) and follow the methods developed in the exploratory trial [25]. The evaluation will take a broad perspective, covering use of all hospital, community health and social services, including complementary therapies, plus productivity losses resulting from time off work or reduced productivity at work due to illness. Data on therapist contacts in the MBCT groups will be collected from therapist records to avoid patients revealing their treatment group to the research assessors. Data on indirect time, including preparation and supervision, will be collected directly from the trial therapists. Data on use of other services will be collected using the Adult Service Use Schedule (AD-SUS). The AD-SUS was developed in several mental health trials and was further modified and successfully employed in the exploratory trial. Productivity losses as a result of time off work or reduced productivity at work due to illness will be measured using the absenteeism and presenteeism questions of the World Health Organization's Heath and Work Performance Questionnaire (HPQ) [47].

The cost of the psychosocial treatments will be directly calculated from salaries using the micro-costing approach employed in the exploratory trial. National UK unit costs will be applied to medication, hospital contacts and community health and social services. Productivity losses will be calculated using the human capital approach, which involves multiplying the individual's salary by reported days off work due to illness, but will be explored further in sensitivity analyses to take into consideration concerns that the human capital approach overestimates these costs [48].

A further element to the economic evaluation will be the estimation of the cost of training of therapists. There are few MBCT therapists in the UK and, should the intervention prove cost-effective, policy makers will require evidence of the investment needed to train new therapists. Detailed information will be collected on the resources that currently go into the MBCT postgraduate training programme at the University of Exeter. These costs will be used to model the longer-term and wider cost impact of MBCT on relative cost-effectiveness.

### Statistical analysis plan

Analyses will be conducted by the trial statistician (RT) following CONSORT standards and overseen by the Data Monitoring and Safety Committee. Initial analyses will be conducted on an intention to treat (ITT) basis, with subsequent analyses being per protocol. All statistical models will be run with and without adjustment for baseline characteristics where the covariate selection is derived from comparison of the treatment groups at baseline.

#### (i) The policy question

For the primary outcome variable, time to relapse/recurrence will be compared using Cox regression proportional hazard survival analysis with treatment condition (MBCT/m-ADM) as the independent variable and allowing for the stratification variables. In a secondary analysis ADM use will be added as an independent variable in the Cox regression model. A per protocol analysis will also be undertaken for the secondary outcomes.

Secondary outcomes will be examined using mixed models Analysis of Variance (ANOVA): between group (ITT/per protocol) and repeated measures (3-24 month follow-ups). Sensitivity analyses will explore the impact of imputation of data losses including loss to follow up and drop out.

#### (ii) The explanatory questions

These will be examined using a mix of three mutually informative methods:

(a) For the MBCT arm time to relapse/recurrence of depression will be compared using Cox regression proportional hazard survival analysis, allowing for the stratification variables and any group differences in ADM tapering/usage (if rates of tapering/usage are different across the two arms).

(b) Mediational analyses will investigate hypothesised mechanisms of change pre-/post-treatment across the trial arms using approaches to testing mediation that allow multiple mediators in one model using the approach set out by Kraemer et al. (2002) and as used in our exploratory trial [49].

(c) Qualitative data will be analysed using thematic analysis (with NVivo software). This will combine inductive and deductive approaches and will be conducted collaboratively by a small sub-group of the research team in order to enhance validity.

The Data Monitoring and Safety Committee will oversee analysis at 3 and 12 months for patient safety and at 12 and 24 months for the primary outcome analyses. Given the public health importance of the findings we anticipate disseminating the findings at 12 and 24 month follow-ups as soon as they are available.

(d) Differences in mean costs will be analysed using standard parametric t-tests with the validity of results confirmed using bias-corrected, nonparametric bootstrapping (repeat re-sampling) [50]. Despite the skewed nature of cost data, this approach is recommended to enable inferences to be made about the arithmetic mean [51]. The primary economic analysis will focus on the policy question: MBCT vs. mADM. Cost-effectiveness will be assessed through the calculation of incremental cost-effectiveness ratios (ICER) and will be explored in terms of relapse/recurrence and quality adjusted life years using the EQ-5D measure of health-related quality of life. Uncertainty around the cost and effectiveness estimates will be represented by cost-effectiveness acceptability curves [52].

### Ethical approval and trial governance

We have received multi-centre ethical approval (South West Research Ethics Committee, 09/H0206/43) and local research governance approval for all sites (NHS Devon covering Exeter and Mid-and North-Devon, PCT0739; NHS Bristol, covering Bristol site, 2010-004, NHS Plymouth and NHS Torbay, PLY-TOR001 covering South Devon site). The study personnel, management group and independent Trial Steering Committee will ensure that the study is conducted within appropriate NHS and professional ethical guidelines, ensuring that Good Clinical Practice guidelines are observed at all times. This trial has been approved by the Medicines and Healthcare products Regulatory Agency (EudraCT number 2009-012428-10). All participants gave full informed consent.

## Competing interests

The authors declare that they have no competing interests.

## Authors' contributions

WK, SB, RB, TD, GL, RT, ERW, PL, DK & NM conceived and designed the study and obtained funding. AE is the lead MBCT therapist responsible for training and supervision. RH is the trial manager. WK drafted the manuscript and all other authors contributed to editing of the final manuscript.
